# Power Reduction with Sleep/Wake on Redundant Data (SWORD) in a Wireless Sensor Network for Energy-Efficient Precision Agriculture

**DOI:** 10.3390/s18103450

**Published:** 2018-10-13

**Authors:** Haider Mahmood Jawad, Rosdiadee Nordin, Sadik Kamel Gharghan, Aqeel Mahmood Jawad, Mahamod Ismail, Mahmood Jawad Abu-AlShaeer

**Affiliations:** 1 Centre of Advanced Electronic & Communication Engineering, Faculty of Engineering and Built Environment, Universiti Kebangsaan Malaysia, UKM Bangi, Selangor 43600, Malaysia; adee@ukm.edu.my (R.N.); aqeel_1986@coalrafidain.edu.iq (A.M.J.); mahamod@ukm.edu.my (M.I.); 2 Department of Computer Communication Engineering, Al-Rafidain University College, Filastin, Baghdad 10064, Iraq; 3 Department of Medical Instrumentation Techniques Engineering, Electrical Engineering Technical College, Middle Technical University, Baghdad, Iraq; sadiq@siswa.ukm.edu.my; 4 Department of Statistic, Al-Rafidain University College, Filastin, Baghdad 10064, Iraq; dean@coalrafidain.edu.iq

**Keywords:** climate conditions, farm field, power consumption, sleep/wake, *SWORD* algorithm, solar cell, WSN, Zigbee

## Abstract

The use of wireless sensor networks (WSNs) in modern precision agriculture to monitor climate conditions and to provide agriculturalists with a considerable amount of useful information is currently being widely considered. However, WSNs exhibit several limitations when deployed in real-world applications. One of the challenges faced by WSNs is prolonging the life of sensor nodes. This challenge is the primary motivation for this work, in which we aim to further minimize the energy consumption of a wireless agriculture system (WAS), which includes air temperature, air humidity, and soil moisture. Two power reduction schemes are proposed to decrease the power consumption of the sensor and router nodes. First, a sleep/wake scheme based on duty cycling is presented. Second, the sleep/wake scheme is merged with redundant data about soil moisture, thereby resulting in a new algorithm called sleep/wake on redundant data (*SWORD*). *SWORD* can minimize the power consumption and data communication of the sensor node. A 12 V/5 W solar cell is embedded into the WAS to sustain its operation. Results show that the power consumption of the sensor and router nodes is minimized and power savings are improved by the sleep/wake scheme. The power consumption of the sensor and router nodes is improved by 99.48% relative to that in traditional operation when the *SWORD* algorithm is applied. In addition, data communication in the *SWORD* algorithm is minimized by 86.45% relative to that in the sleep/wake scheme. The comparison results indicate that the proposed algorithms outperform power reduction techniques proposed in other studies. The average current consumptions of the sensor nodes in the sleep/wake scheme and the *SWORD* algorithm are 0.731 mA and 0.1 mA, respectively.

## 1. Introduction

Precision agriculture (PA) is a supervision procedure that uses information technology to improve crop production and quality. The use of wireless sensor networks (WSNs) in agriculture to monitor climate conditions and to provide farmers with a considerable amount of information has been considered. WSNs cannot be deployed easily because they represent the security and economy of countries. The potential applications of WSNs in the civilian domain include agricultural [1], industrial [2], healthcare [3], natural disaster [4], and monitoring uses. In recent years, WSNs have been extensively used in various monitoring applications for PA. WSN technology presents many advantages, such as low cost, scalability, reliability, accuracy, flexibility, low power requirement, and easy deployment, which enable its use in diverse applications [5,6,7]. PA is a control scheme that utilizes information technology to improve crop quality and production. It is an advanced technology for enhancing crop production in different types of farm. PA is widely adopted to minimize diseases and pests, and consequently, reduce the use of pesticides, thereby leading to efficient and environmentally acceptable agriculture [7,8]. Having a similar routine and yield regardless of locations, conditions, and labor intensity can be avoided through PA control. The most important applications of PA in agriculture are in the monitoring of air temperature, relative humidity, soil moisture, and vapor pressure deficit to reduce production risks prior to the cultivation of a specific crop [9,10]. 

The advancement of WSNs has produced new research approaches in agriculture. Furthermore, the development of microelectromechanical technologies has resulted in the manufacture of small and low-cost sensors. The extensive use of microcontrollers and microprocessors, which involve small, self-modifiable sensor nodes, low-cost tools, and scalability, indicates that WSNs can be used in agriculture computerization [5]. However, a number of limitations and challenges must be addressed before WSNs can be utilized to monitor diverse agricultural environments. The primary limitations and challenges in current PA applications that rely on WSNs have been highlighted. Moreover, proposals on how to address them have been provided.

The first challenge is extending the battery life and reducing the power consumption of sensor nodes. Reducing the power consumption of radio frequency (RF) modules considerably minimizes the power consumption of sensor nodes because RF modules use considerable power [11]. The power consumption problem can be resolved by adopting a specific power reduction technique or algorithm. An energy-harvesting technique can also be combined with the selected power reduction technique.

The second challenge is communication distance. WSNs suffer from the effects of harsh ecological environments due to their wide communication area in agricultural fields [12]. Consequently, data packets are lost in the transmitting and receiving processes [6,13]. In agricultural applications, however, when distance increases between nodes in the network due to the large width of agriculture fields, the communication range can be lengthened by considering different network topologies, such as mesh networks. Mobile router nodes, such as unmanned aerial vehicles or drones, can also be utilized. Drones can be used to pass data from sensor nodes to the coordinator node via a multihop process. However, using drones results in other limitations.

The third challenge is the consideration of cattle tracking and localization in smart farms based on WSNs [14]. WSNs are used to monitor animal behavior and positions, such as standing, walking, grazing, and lying down [15,16]. In this context, several problems, such as animal situation and mobility, radio interference, changes in network topology, height of animal collar, signal penetration depth over the animal body, and antennas of access points, can arise and must be considered [17]. Robust tracking and localization techniques, such as smart tracking and localization that rely on intelligent techniques or optimization algorithms, can be adopted to overcome these challenges.

The fourth challenge is propagation losses. When WSNs are used in agricultural applications, they must operate in different environments and climate conditions, such as bare land, ground, greenhouses, orchards, complex topography, and farms. These surroundings affect radio propagation performance. Consequently, simple or complex communication topologies in WSNs suffer from serious challenges. Transmission from one sensor node to another in agricultural application requires crossing over dense crops, where the clearance communication channel cannot be ensured. Therefore, communication link quality must be guaranteed when deploying WSNs. The performance of WSNs is associated with the working environment. Hence, an accurate modeling of wireless communication channel path loss must be established to represent propagation features. A path loss model can provide precise optimization and performance evaluation of a network during the distribution design process to improve the power consumption of nodes [18] and enhance localization and target detection [16], thereby ensuring the quality of services and minimizing the number of retransmission events in a network [19]. Reference [13] provided abundant information on the challenges and limitations of WSN deployment in agricultural application.

The first challenge, namely, reducing the power consumption and extending the battery lifetime of sensor and router nodes in agricultural application, is addressed in the current study. Several energy-efficient methods and solutions, such as power reduction and energy-harvesting techniques, have been presented in the previous literature to solve the power consumption problem of sensor nodes. Among these solutions, the sleep/wake scheme and the combination of sleep/wake and data mitigation (redundant data) described as the sleep/wake on redundant data (*SWORD*) algorithm in this study are considered to minimize energy consumption and increase the lifetime of nodes in WSNs. Battery-operated radio modules consume the most power in sensor and router nodes. Therefore, the energy consumed by sensor nodes can be improved. Furthermore, battery life can be extended by reducing the amount of communication and minimizing redundant data in a network via the *SWORD* algorithm. Accordingly, the deployed nodes are expected to work for months or years [20]. In addition, WSNs can be equipped with solar energy (based on a solar cell) to supply nodes with energy and to charge their batteries.

The novelty of this study lies in the merging of the sleep/wake scheme with the redundant data on soil moisture, thereby resulting in a new algorithm, namely, *SWORD*. The *SWORD* algorithm can considerably minimize the power consumption and data communication of a sensor node to 0.1 mA and 86.45%, respectively, relative to traditional operation. In addition, based on our literature review published previously in [13], we discovered that each power reduction techniques used in agricultural applications only relies on one method such as cluster a head, sleep/wake, data mitigation, transmission power control, data compression, data driven, routing protocol, and sink mobility schemes. A small number of these methods are merged into two or more power reduction techniques [21,22,23,24] to further reduce the power consumption and extend the battery life of sensor nodes. However, none of these studies has focused on an approach similar to SWORD, particularly in the case of PA. The other contributions of this work are summarized as follows.

(i) The power consumption of the sensor and router nodes of a wireless agriculture system (WAS) is modeled.
(ii) The power consumption and data communication of the adopted WAS are minimized, and battery life is prolonged using two power reduction techniques, namely, the sleep/wake scheme and the *SWORD* algorithm. Considerable power saving is achieved by the WAS when the proposed *SWORD* algorithm is used.
(iii) Our results are compared with those of similar studies in terms of power consumption to verify the performance and efficiency of the proposed sleep/wake scheme and *SWORD* algorithm.

## 2. Related Studies

Researchers have developed several power reduction techniques in their search for an infinite power source for sensor nodes to achieve a limitless life span. This section presents several WSN power reduction methods that can be used in PA. Early studies on energy-efficient PA can be traced back to Zhu et al. [6], who developed a WSN based on an agricultural monitoring system. They discovered that the effective communication distance between nodes is more than 200 m in an open-field environment and the average packet loss rate is 7.6%. These authors adopted a sleep/wake algorithm to reduce the power consumption of the WSN. The received power became attenuated and sinusoidal when the distance between a transmitter and a receiver was increased. The sensor node woke up for 30 s every 4.5 min. The power consumption of the sensor node relative to that in traditional operation (i.e., 80 mA) was 53 mA. A power saving of 33.75% was achieved. Zou et al. [25] proposed methods to optimize data transmission and to extend network life by using a prediction algorithm for the energy harvested from a solar cell via environmental shadow detection. The mechanisms sustained network activities in an uninterrupted and efficient manner in the experimental study. However, a solar cell system is generally irregular and extensively influenced by weather changes. The power consumption of a sensor node in conventional operation is 80 mA, whereas power consumption is minimized based on the duty cycle (DC), which is set to a fixed value. The peak current consumption of the sensor node is 4.5, 18, and 20 mA, which corresponds to 10% DC in empty mode, 30% DC in lacking mode, and 100% DC in sufficient mode, respectively. Srbinovska et al. [20] collected environmental parameter data from distributed WSNs. They adopted a sleep/wake strategy to reduce the power consumption of the sensor node in a WSN. The current consumption of the sensor node is minimized to 142 µA relative to that in traditional operation, which consumes 24 mA, by switching between sleep and active modes in low DC.

Nguyen et al. [26] observed the impacts of climate change on crop fields by using WSNs. The authors adopted a sleep/wake algorithm to reduce the power consumption of WSNs. The data collection method improved the power consumption of the network. The advantages are low cost and ubiquitous monitoring. Moreover, the system can be widely applied to agriculture in developing countries. The Zigbee wireless protocol was configured to operate for 30 s every 15 min (i.e., DC equal to 3.3%). Therefore, the 129 mA power consumption of the sensor node in traditional operation is reduced to 17.25 mA when the DC strategy is adopted. Eto et al. [27] charged batteries via solar energy generation to solve the problem of covering an agricultural field with mobile sensor nodes. The results exhibited a 4% reduction in the number of nodes and a 10% extension of operation lifetime compared with the conventional method. The selection of the leader node was performed by calculating residual energy. In this case, the power consumption of the node can be extended to 90 working days (i.e., consumes 11.11 mA at a battery capacity of 1000 mAh). Fourie et al. [28] designed a fish pond management system for fish conservation by using an autonomous solar-powered system. Zigbee can enter into sleep mode to improve the power consumption of WSNs. The results indicated that the system can successfully control the pond’s temperature and dissolved oxygen level. The use of a maximum power point tracking (MPPT) charging controller allows the platform to utilize energy higher than 8 W. However, the components of the system, including a solenoid valve, a stepper motor, and a sensor node hardware, consume a high amount of power, i.e., approximately 4 W. Meanwhile, the sensor node components (i.e., sensor, processor, and RF module) exhibit a low power consumption of approximately 207 mW relative to the total consumption. Therefore, the current consumption of the sensor node is 81.5 mA. When a solar cell with DC charging is adopted, the power efficiency is improved by 31%. However, power efficiency increases to 98% when MPPT is used.

Bapat et al. [29] developed a WSN application for crop protection from animal intrusion in a farming field. A sleep/wake scheme was adopted to conserve the power of the WSN. Successful results were obtained from laboratory-level trials of the systems. However, the authors discovered a few technical faults in the circuit components. The power consumption of the system was 27 W/h, but the calculations of the current consumption of the nodes in the WSN were not considered in their study. Villarrubia et al. [30] constructed practical organizations of agents that can connect with one another while observing crop irrigation. Fuzzy logic was adopted to accurately control watering quantities. The major finding of this research was that heterogeneous data from the surrounding can be merged via sensor node measurements. The proposed system based on fuzzy logic consumes 4.5 L of water per day relative to traditional operation, which consumes 7.3 L a day. Thus, 37% water saving is achieved over 30 days. The use of a solar cell also allows the continuous charging of the sensor node battery. Navarro-Hellín et al. [31] monitored soil water status and irrigation water by developing a practical application to optimize water resources in irrigated agriculture. They adopted a sleep/wake scheme to reduce the power consumption of the General Packet Radio Service (GPRS) node. The sending and sampling rates in the achieved tests were adjusted to 30 min and 15 min, respectively. The disadvantage of this system is its short life span of 13.35 days. However, the average current consumption of the entire GPRS node is 5.93 mA relative to that in traditional operation, where the GPRS modem alone consumes 400 mA during data transmission.

De la Concepción et al. [24] presented an efficient WSN platform that is appropriate for agricultural applications and can be used in remote areas. A sleep/wake algorithm was proposed to limit the energy consumption of the WSN platform. The results showed that the nodes are autonomous, scalable, and easy to locate and relocate. However, the proposed system has a limited communication range because the transceiver uses an omnidirectional antenna. Nevertheless, the sensor node captures and transmits an image for 6 min and then sleeps for 54 min, with a low power consumption of 0.8 µA. Subsequently, sensor node life is extended to 8 days. Moreover, current consumption is improved to 4.427 mA compared with that in conventional operation, where the current consumption of the camera and CC1110 is 270 mA and 31 mA, respectively, in the transmission mode. Cambra et al. [32] discovered, examined, and auto-calibrated imbalances in the pH levels of the nutrient solution used in hydroponic agriculture. The results indicated that the design of the system improved water quality in hydroponic agriculture by implementing and testing a smart system for bicarbonate control in irrigation. The advantages include low power consumption, low cost due to the use of a low-power wireless protocol (i.e., nRF24L01) while ensuring the adopted coverage area, and the provision of multimedia services because of the adoption of mobile devices or computers. However, the number of nodes in the network is limited to five. Each node wakes up for 15 s to transmit their data and then returns to sleep for 255 s (i.e., DC is equal to 15/270 = 5.555%). Accordingly, the power consumption of the sensor node is reduced from 49 mA (in traditional operation) to 2.807 mA (with the sleep/wake strategy).

Ilie-Ablachim et al. [33] monitored environmental parameters for greenhouse applications. They used the MoniSen module to introduce a new granularity level for PA monitoring applications. They adopted a distributed sensor architecture for large-scale applications by developing fully functional software and hardware. The sleep/wake algorithm was applied to reduce power consumption. Moreover, the sensor node in the system was configured to transmit data for 48 s every 24 h (i.e., DC is equal to 3.3%). Consequently, battery life is prolonged to 4408 h. The total current consumption of the system is 0.544 mA relative to that in traditional operation, where the sensor node consumes 46.145 mA with a power efficiency of more than 90%. Zaier et al. [34] tested and developed a smart irrigation system based on WSN and solenoid electrovalves. The smart irrigation system was implemented in 14 farms. The power consumption of the WSN was improved by the proposed sleep/wake strategy by using periodic sleeping cycles. The power consumption of the system is extremely low in sleep mode. The power consumption of the XBee Pro S2 module of the sensor node is 177 mA in active mode, 3.5 µA in sleep mode, and 7 mA in active mode for the soil moisture sensor. Power consumption is improved and battery life is extended through the power-down mode of XBee.

All the aforementioned studies are summarized in Table 1. The table highlights the improved power consumption and the related power reduction technique or scheme in each work. The table also provides the hardware, including wireless protocol, microcontroller or processor, climate condition sensors, battery type and capacity, and power and voltage of the solar panel, adopted in each study. The future of the agriculture industry can be revolutionized by relying on computerized systems, advanced sensors, and energy-efficient wireless networks instead of using the traditional agriculture system that is proven to be inefficient, labor intensive, and has low productivity.

## 3. WSN Topology

**Figure 1:**
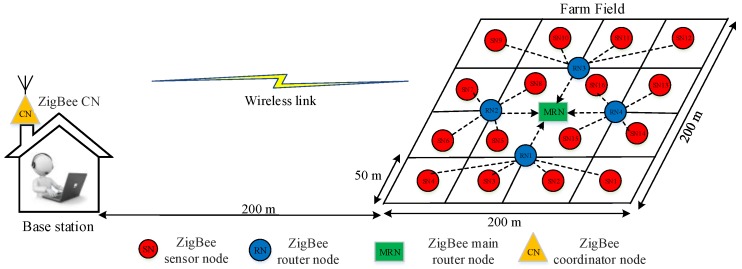
WSN topology of the proposed WAS for precision farming.

In this work, we aim to reduce unnecessary energy consumption and to prolong the battery life of the network, along with the sensor and router nodes that are distributed across a farm field. Figure 1 shows the proposed topology of the wireless nodes in the farm field with a layout of 200 × 200 m^2^, which represents the available tested area in this study. This area can be potentially extended to actual medium-size commercial crop farm areas. The farm field consists of 16 sensor nodes, four router nodes, the main router node, and the coordinator node. Each sensor node is responsible for collecting climate conditions, such as air temperature, air humidity, and soil moisture, from a square area of 50 × 50 m^2^. The sensor node is located at the center of the square area. Four sensor nodes communicate wirelessly to one of the router nodes. The collected data (i.e., climate conditions) from the sensor nodes are transmitted wirelessly to the central router via the router nodes.

The main router node passes the climate conditions to the coordinator node, which is located at the base station (farmhouse). Our proposed topology considers only fixed router nodes for communication between sensor nodes and the coordinator node. In addition, the changing roles of the router nodes are not considered, as shown in Figure 1, because the focus of this study is the physical layer approach for achieving energy efficiency, whereas changing roles involve higher layers, such as media access control (MAC) and network. Thus, the changing roles can provide a future opportunity to continue the current study to further understand the benefits of energy savings based on cross-layer dimensions.

The distance between the farmhouse and the crop field is approximately 200 m. Therefore, an appropriate wireless communication protocol, such as Zigbee (XBee S2C), must be used to ensure data delivery. This module can theoretically guarantee 1.2 km in outdoor environments [38], low power consumption, low cost, and battery operation in WSNs. In terms of power consumption, Zigbee (XBee S2C), which consumes 36.9 mW as reported in [13], is better than LoRa, SigFox, WiFi, and GPRS, which consume 100, 122, 835, and 560 mW, respectively. Nevertheless, the current work can be extended to cater to future wireless technologies, including LoRa.

## 4. Hardware Configuration of the Proposed WAS

**Figure 2:**
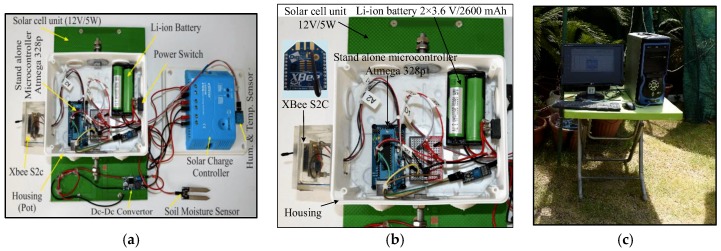
Hardware of the WAS: (**a**) sensor node, (**b**) router node, and (**c**) coordinator node.

The range covered by the individual node and the total area of the crop field can be set based on the number of nodes to be deployed in the field. Table 2 presents the hardware for the sensor node of the proposed WAS. Figure 2 shows the hardware of the sensor, router, and coordinator nodes. It presents a simple hardware implementation of the WAS, which involves minimal interface and connection between sensors to the microcontroller and the wireless links. The digital humidity–temperature (DHT11) sensor and the soil moisture sensor require only three wires for a physical interface with the microcontrollers: (i) supply voltage, (ii) ground, and (iii) output data. The first two wires are used to power the sensors by +5 V, whereas the third wire is the analog and digital serial output signals for the soil moisture sensor and DHT11, respectively. The microcontroller of the sensor and router nodes communicates with the Zigbee wireless protocol through a single-wire data bus.

**Figure 3:**
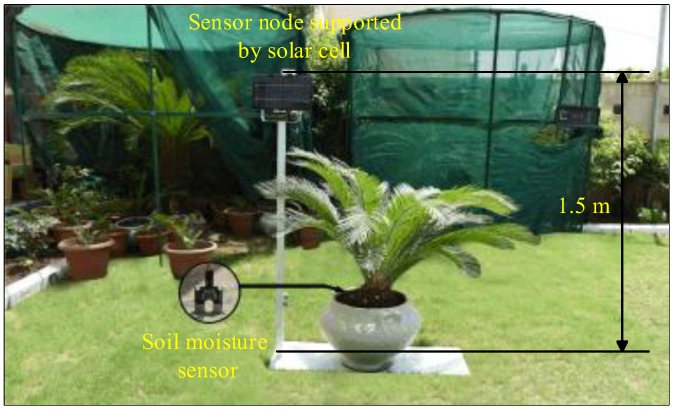
Sensor node installed at the farm.

The sensor and router nodes are fixed on a surface that is 1.5 m above the ground (Figure 3), which is the recommended height in [39], to avoid the effect of the Fresnel zone or signal reflection. The sensor and router nodes in the tested area are powered by a battery. The coordinator node is connected to a laptop/PC in the base station. Therefore, this node is unaware of the energy supply. The PC is equipped with graphical user interface (GUI) software to monitor the climate conditions in the farm field. The sensor and router node structures are designed to work day and night. At daytime, the components, sensors, microcontroller, and XBee S2C are powered by solar energy. Furthermore, a solar cell provides energy to charge the batteries of the sensor and router nodes. The solar panel is positioned in front of the sensor node box at a low inclination angle (20°–30°) to orient the solar cell panel relative to the sun. In addition, two power reduction algorithms (i.e., sleep/wake and *SWORD*) are adopted to further reduce the power consumption of the sensor and router nodes. At nighttime, the components only use battery energy as supported by the two power reduction algorithms. For WSN simplicity and to reduce the complexity of the proposed WAS, one sensor node, one router node, the main router node, and the coordinator node are practically implemented to monitor the climate conditions in the farm field.

## 5. Zigbee Data Packet Length

The transmitted data packets for the sensor, router, and main router must be determined to identify the time consumption of the XBee S2C wireless module. The active transmission time (*t_TX_*) of XBee S2C, which is based on the Zigbee wireless protocol, can be expressed as [42]:

(1) $$t_{TX}=t_{SA}+\frac{L}{D}\;,$$

where *t_SA_* is the transient time of XBee S2C from sleep mode to active mode. XBee S2C consumes 10.2 ms when pin sleep is used [43]. *L* denotes the data packet length of XBee S2C in bits, and *D* indicates the XBee S2C data speed of 250 kbps for the XBee S2C module using the 2.4 GHz industrial, scientific, and medical frequency band.

**Figure 4:**
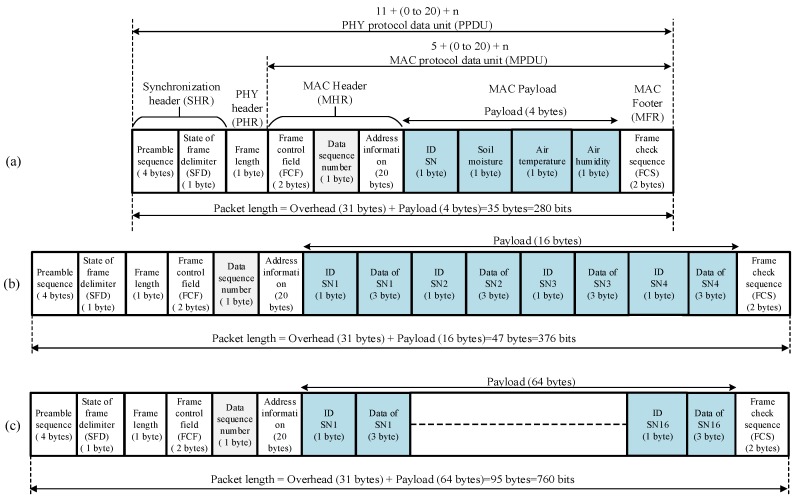
Data packet of XBee S2C is compatible with the Zigbee protocol [44]: (**a**) data packet of each sensor node (35 bytes), (**b**) data packet of each router node (47 bytes), and (**c**) data packet of the main router node (95 bytes).

The data packet length consists of 31 bytes (overhead) and 35, 47, and 95 bytes (payload) for the sensor, router, and main router nodes (shown in Figure 4a–c, respectively). In the current work, the overhead bytes are constant. Moreover, the data bytes differ for the sensor, router, and main router nodes, which rely on the parameters of climate conditions. The data packet length and the active transmission time of the adopted nodes can be described as follows:

1. *Sensor node*: The data packet length of each sensor node consists of 35 bytes (i.e., 280 bits). The payload includes 4 bytes, namely, (i) identification (ID) of the sensor node, (ii) air temperature data, (iii) air humidity data, and (iv) soil moisture data (Figure 4a). Therefore, the active transmission time for each XBee S2C in the sensor node based on Equation (1) is 11.32 ms.
2. *Router node*: The data packet length of each router node consists of 47 bytes (i.e., 376 bits). The payload includes 16 bytes, i.e., 4 bytes for each sensor node, where each router node collects data from four sensor nodes (Figure 4b). Therefore, the active transmission time for each XBee S2C in the router node based on Equation (1) is 11.704 ms.
3. *Main router node*: The data packet length of the main router node consists of 95 bytes (i.e., 760 bits). The payload includes 64 bytes, i.e., 16 bytes for each router node, where the main router node collects data from four router nodes (Figure 4c). Therefore, the active transmission time for XBee S2C in the main router node based on Equation (1) is 13.24 ms.

The data packet length of XBee S2C includes a maximum of 127 bytes (31 bytes overhead and 96 bytes payload) [45]. Therefore, the adopted Zigbee wireless protocol using XBee S2C is adequate to achieve the proposed WSN topology in the current work, as presented in Figure 1. The maximum data packet length of 95 bytes is used to monitor the climate conditions of the agricultural field.

Delay in WSNs denotes the difference between the time when the information is produced in the sensor node and the time when the information arrives at the sink node. Being delay-sensitive poses a challenge to WSNs, particularly with multihop counts [46]. Most information is delay-tolerant in the majority of agriculture applications [47,48]. In addition, a small proportion of delay-sensitive information exists. Being delay-sensitive is essential when multihops are used in WSNs and the data is required to be monitored in real time. In our application, however, climate conditions (i.e., temperature, humidity, and soil moisture) are delay-insensitive because the data are collected and transmitted from the sensor node to the coordinator node through a single router node. Evidently, delay is not a major concern in such PA case. Therefore, delay is not considered in the current study.

## 6. *SWORD* Algorithm

**Figure 5:**
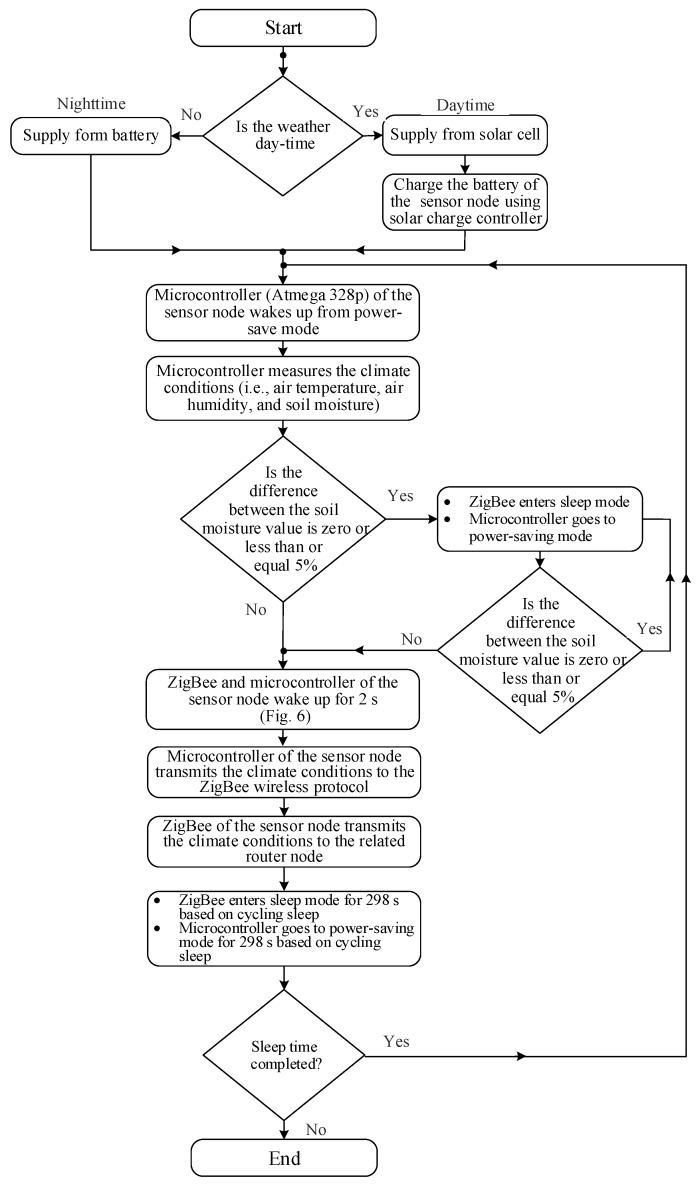
Flow diagram of the *SWORD* algorithm.

**Figure 6:**
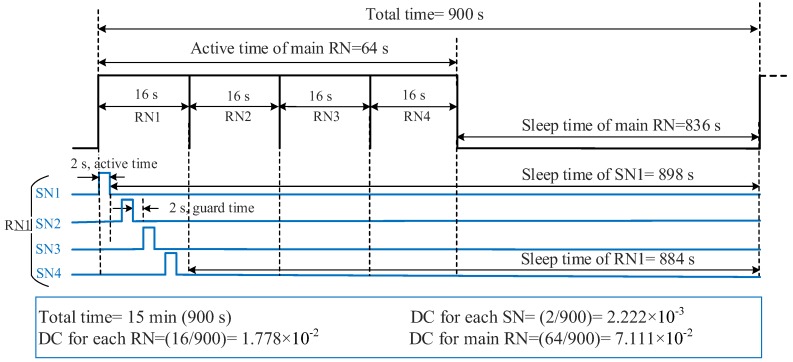
Timing diagram of the sleep/wake scheme of the sensor and router nodes.

The *SWORD* algorithm (Figure 5) is designed and implemented in the microcontroller Atmega 328p of the sensor node to reduce power consumption. The *SWORD* algorithm proceeds as follows:

1. The microcontroller Atmega 328p initially wakes up from sleep mode.
2. All the components of the sensor node (i.e., sensors, microcontroller, and XBee S2C) are supplied with energy from a solar cell (12 V/5 W) at daytime to charge their batteries. The batteries are used to supply power to the sensor node at nighttime.
3. The microcontroller measures the climate conditions (i.e., air temperature, air humidity, and soil moisture).
4. The microcontroller measures the difference between the previous and subsequent soil moisture values (soil moisture difference = |previous value − subsequent value|) to check whether redundant data exist.
5. When the difference between the two values is zero or less than or equal to 5% (threshold level), the sensors and the XBee S2C module enter sleep mode. Furthermore, the microcontroller goes to power-saving mode to save the energy of the sensor node. In such case, the soil is wet and no data are transmitted from the sensor node to the router node. In addition, irrigating the soil is unnecessary, thereby leading to water saving. The components of the sensor node remain in sleep mode until the threshold level is exceeded. A slight difference in value of 5% is selected to obtain a precise decision. In agriculture, irrigation systems depend on soil moisture measurements, wherein soil is considered a crucial part of planning tools for conducting irrigation [49,50]. When soil is dry, the irrigation system is operating; otherwise, the irrigation system is off (i.e., wet soil). Therefore, the *SWORD* algorithm is determined on the basis of soil moisture measurement in the current study. By using soil moisture, irrigation is scheduled to sustain soil moisture conditions equivalent or close to the field capacity to satisfy the required crop water requirements. In addition, several sensors are being considered for future work to capture relevant parameters related to agriculture, such as soil temperature, soil conductivity, salinity, leaf wetness, and rainfall sensors.
6. By contrast, all the components of the sensor node remain awake when the difference between the two values is greater than 5%. Therefore, the measured data in Step 3 are transmitted from the sensor node to the coordinator node via the router nodes. After the transmission process is completed, the sensors and XBee S2C module enter sleep mode. Furthermore, the microcontroller goes to power-saving mode to save the energy of the sensor node. The sensor node transmits the measured data about climate conditions every 15 min (900 s) for 2 s (i.e., extremely low DC 2/900 = 2.222 × 10^−3^). The sensors require 1 s to measure the data on climate conditions. In addition, another second is required to transmit the data to the related router node, including the active transmission time of XBee S2C (i.e., 11.32 ms, as shown in the previous section) and the microcontroller. This situation indicates that 2 s are consumed by each sensor node to measure and transmit climate conditions to the related router node, as shown in the timing diagram in Figure 6. Consequently, each sensor node wakes up for 2 s and sleeps for 898 s.
7. The four sensor nodes communicate using one router node (Figure 1). Therefore, the allocated time for each router node is 16 s. From the 16 s, 8 s is for the four sensor nodes, whereas 8 s is for the guard time (2 s after data transmission of each sensor node to avoid data collision), as shown in Figure 6. The router nodes, such as RN1, RN2, RN3, and RN4, collect and transmit the data of the four sensor nodes to the main router node within 16 s and then enter sleep mode. Consequently, each router node wakes up for 16 s and sleeps for 884 s (i.e., extremely low DC 1.778 × 10^−2^), as shown in Figure 6.
8. The main router node gathers the collected data of the four router nodes and transmits these data to the coordinator node within 64 s. The main router node then enters sleep mode for 836 s after data transmission (i.e., DC is 7.111 × 10^−2^), as shown in Figure 6.

## 7. Power Consumption Models

### 7.1. Sensor Node Power Consumption Model

The life span of sensor nodes is the time that lapsed from the first transmission until the sensor nodes lose their sensing capability. The life span of a WSN relies on the current consumed by each node in the network. The power consumption of sensor nodes depends on the number of components. In this study, the sensor nodes include air temperature and humidity sensors embedded into the DHT11 sensor, a soil moisture sensor, the Atmega 328p microcontroller as a standalone system for reducing power consumption, and the XBee S2C wireless protocol. Among the sensor nodes, XBee S2C is considered the main power consumer [51]. When the consumption values of these components are added, the total current consumption of the sensor node can be expressed as Equation (2):

(2) $$I_{SN}=\;I_{Soil}+\;I_{DHT}+I_{Atmega}+I_{XBee\;S2C},$$

where *I_Soil_* is the current consumed by the soil moisture sensor, *I_DHT_* is the current consumed by the air temperature and humidity sensors, *I_XBee S2C_* is the current consumption of the XBee S2C wireless technology, and *I_Atmega_* is the current consumed by the Atmega 328p microcontroller.

The measurements of each sensor node component were achieved by using a storage oscilloscope (MCP Lab Electronics/DQ7042C) and a digital multimeter (MCP Lab Electronics/MT8045) to evaluate the current consumption of the sensor node. Therefore, the current drawn by the sensor node is presented for two cases. The first case, which is a conventional operation (i.e., without a sleep/wake scheme or any power reduction technique), is formulated in Equation (2). The second case, which involves a sleep/wake scheme, can be expressed in terms of average current consumption, as presented in Equations (3)–(7):

(3) $$I_{Soil\_avg\;}=\mathrm{DC}\;\times \;I_{Soil\_active}+\left(1-\mathrm{DC}\right)\;\times \;I_{Soil\_sleep},$$

(4) $$I_{DHT\_avg\;}=\mathrm{DC}\;\times \;I_{DHT\_active}+\left(1-\mathrm{DC}\right)\;\times \;I_{DHT\_sleep},$$

(5) $$I_{ATmega\_avg\;}=\mathrm{DC}\;\times \;I_{ATmega\_active}+\left(1-\mathrm{DC}\right)\;\times \;I_{ATmega\_sleep},$$

(6) $$I_{XBee\;S2C\_avg\;}=\mathrm{DC}\;\times \;I_{\mathrm{XBee}\;S2C\_active}+\left(1-\mathrm{DC}\right)\;\times \;I_{\mathrm{XBee}\;S2C\_sleep},$$

(7) $$I_{SN\_avg\;}=I_{Soil\_avg\;}+I_{DHT\_avg\;}+I_{ATmega\_avg\;}+I_{XBee\;S2C\_avg\;},$$

where *I_avg_* is the average current consumption of each sensor node component. DC is the proposed duty cycle of the sensor node, which provides an effective method for achieving energy efficiency and can be computed using the ratio of the active time to the total time (*t_active_/T_total_*). DC is configured to 2.222 × 10^−3^ to reduce the power consumption of the sensor node, where the active time is 2 s, the sleep time is 898 s, and the total time is 15 min (900 s). *I_active_* and *I_sleep_* are the active and sleep current consumption of each component of the sensor node.

The overall power consumption of the sensor nodes (*I_SN_total_*) without t sleep/wake scheme can be expressed as Equation (8);

(8) $$I_{SN\_total}=\;\sum_{1}^{n}I_{SN_{n}}\;,$$

where $I_{SN_{n}}$ is the current consumption of each sensor node. In addition, *n* is the number of sensor nodes, and *n* = 16 in the current work.

Given that DC is equal in each sensor node, the total current consumption ($I_{SN\_avg\_total}$) of the sensor nodes can be calculated based on Equation (9):

(9) $$I_{SN\_avg\_total}=\sum_{1}^{n}I_{SN_{n}\_avg}\;,\;$$

where $I_{SN_{n}\_avg}$ is the average current consumption of each sensor node that is modeled in Equation (7).

The average power consumption (*P_avg_SN_*) of a sensor node can be calculated by multiplying the average current consumption with the supply voltage of the sensor node (i.e., 2 × 3.7 V = 7.4 V), as shown in Equation (10). The sensor node life span (*L_life_*) based on the sleep/wake scheme can be expressed as Equation (11):

(10) $$P_{average\_SN}=I_{avg\_SN}\;\times \;V,$$

$$L_{life}=\frac{C_{Battery}}{I_{SN\_avg}}$$

where *C_Battery_* is the initial battery capacity of the sensor node in mAh. In the current work, two Li-ion rechargeable batteries (7.4 V/2600 mAh) are used to supply each sensor node with power.

### 7.2. Router Node Power Consumption Model

In this work, each router node consists of an Atmega 328p microcontroller and the XBee S2C wireless protocol. Therefore, the power consumption of the router and main router nodes depends only on these two components. The power consumption of the router and the main router nodes without and with the sleep/wake scheme can be expressed as Equations (12) and (13), respectively:

(12) $$\;I_{RN}=I_{Atmega}+I_{XBee\;S2C}\;$$

(13) $$\;I_{RN\_avg\;}=I_{Atmega\_avg\;}+I_{XBee\;S2C\_avg\;}\;$$

The total current consumption of the router nodes without and with the sleep/wake scheme can be expressed as Equations (14) and (15), respectively:

(14) $$I_{RN\_total}=\;\sum_{1}^{m}I_{RN_{m}}\;,$$

(15) $$I_{RN\_average\_total}=\sum_{1}^{m}I_{RN_{m}\_average}\;,$$

where $I_{RN_{m}}$ is the current consumption of each router node, as presented in Equation (12); $I_{RN_{m}\_average}$ is the average current consumption of each router node, as modeled in Equation (13); and *m* is the number of router nodes, where *m* = 4 in the current work. Equations (10) and (11) can be utilized to calculate the average power consumption and life span of the router and main router nodes based on the sleep/wake scheme, respectively.

### 7.3. Energy Harvesting Techniques

Energy scavenging or harvesting involves a massive amount of energy from different environments, such as thermal, solar, vibration, and wind. Energy harvesting approaches are effective in improving network life span [52]. Among various forms of environmental energy, solar cell energy is selected in the current work to supply power to the sensor, router, and main router nodes. In energy harvesting, a battery is cyclically recharged to preserve the life span of nodes that continuously operate in the network rather than focusing on reducing energy depletion. However, an energy-harvesting platform must be incorporated into the network to efficiently use the harvested energy.

The current consumption of the sensor nodes from the battery depends on the type of application [53]. In our work, the solar panel is positioned in front of the sensor node box. The solar cell angle is oriented toward the sun with an incident angle of 20°–30° relative to the ground [8]. KINGRO-004V solar cell (KINGRO, Shaoxing, China), which is a first-generation polycrystalline solar cell (12 V/5 W) that delivers a maximum current of 416 mA, is selected (Figure 3). Table 3 provides the characteristics of the adopted solar cell.

Mathematical models should be created for the battery and solar cells of the sensor node to investigate the harvested energy from the solar cell and battery consumption. The power consumption model of the sensor, router, and main router nodes based on the battery is presented in Equations (2)–(15), as shown in Section 7.1 and 7.2. The solar cell is formulated in the current section. Solar cell efficiency (*ƞ*) can be expressed as [25]:

(16) $$\eta_{solar}=(P_{max}/S\;\times \;R),$$

where *P_max_* is the output power of the solar cell (measured in W); *S* is the solar cell surface area (measured in m^2^); and *R* is the radiation, which can be defined as the intensity of the incident light power on the surface of a solar cell (measured in W/m^2^).

## 8. Calibration of Sensors

**Figure 7:**
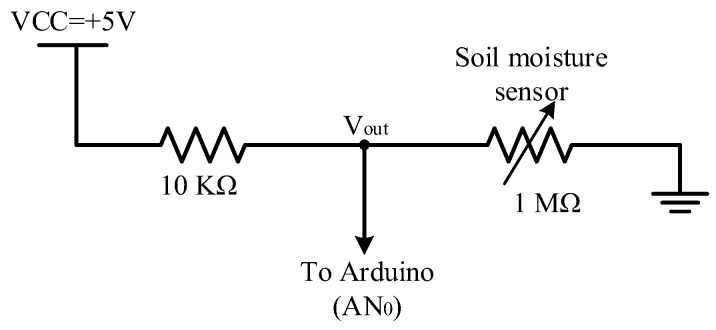
Circuit diagram of the soil moisture sensor.

**Figure 8:**
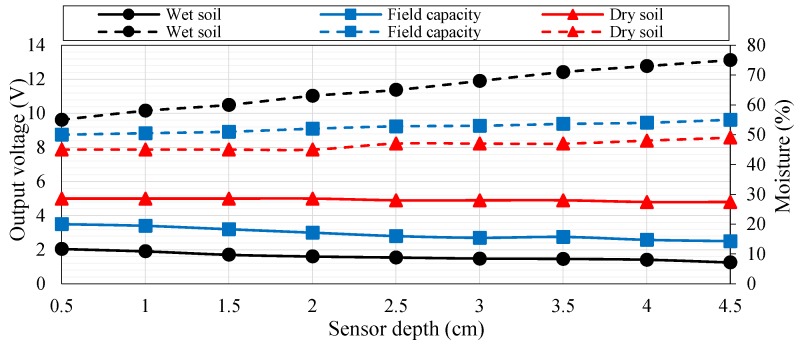
Calibration of the soil moisture sensor.

Figure 7 shows the connection of the soil moisture sensor to the microcontroller (connected to Pin ANo). The soil moisture sensor is connected to a 10 KΩ resistance as a voltage divider, as shown in Figure 7. The middle point (i.e., *V_out_*) between the soil moisture sensor and the resistor is used for sensing variations in output voltage from the changes in moisture value. The output voltage (*V_out_*) is altered between 5 and 0 depending on the moisture of soil. The moisture corresponds to the analog-to-digital converter of the Arduino microcontroller 0–1023 (10 bit resolution). The output voltage of the voltage divider can be translated into moisture in percentage through the microcontroller algorithm. The resistance of the soil moisture value decreases with increasing soil moisture. Therefore, the output voltage decreases. By contrast, the resistance of the soil moisture value increases when soil is dry, thereby increasing output voltage. The soil moisture sensor is calibrated in-field, as shown in Figure 8. The calibration of the soil moisture sensor (Figure 8) is achieved through an experimental test of three cases: (i) wet soil, (ii) field capacity soil moisture, and (iii) dry soil for soil mixture (clay and sandy soil). This calibration can be used in all seasons. The figure shows that the output voltage values of the soil moisture sensor are plotted on the left *y-*axis, whereas the moisture values in percentage are plotted on the right *y-*axis with respect to the depth of the soil moisture sensor. However, the values of the moisture percentages that are presented in Figure 8 are used in the microcontroller algorithm to determine the threshold level between dry soil and wet soil. From these values, the soil moisture sensor achieves self-calibration before each new measure based on the stored data in the algorithm of the microcontroller to reduce measurement errors.

The temperature and humidity sensor (i.e., DHT11) was calibrated to be extremely precise by the manufacturer in a laboratory [54]. The calibration coefficients of the sensor are stored as a program in the one-time programmable (OTP) memory. When the OTP memory is programmed, the contents cannot be changed and are retained after power is turned off. This sensor contains a negative temperature coefficient component for temperature measurement and a resistive component for humidity measurement. DHT11 is a digital sensor that can be connected to an 8 bit microcontroller using a single wire serial interface. It provides fast response, and exhibits high-quality, cost-effective, and anti-interference ability. Consequently, the DHT11 sensor does not require a calibration process and can work accurately to provide air temperature and humidity readings in any seasons.

## 9. Results and Discussion

### 9.1. Current Consumption Measurements

The power consumption of the WAS is a combination of several hardware components, namely, air temperature and humidity (DHT11) sensor, soil moisture sensor, a standalone Atmega 328p microcontroller, and the XBee S2C module. The router and main router consist only of the standalone Atmega 328p microcontroller and the XBee S2C module. The current consumption of the sensor, router, and main router nodes is measured on the basis of three cases, namely, (i) traditional operation (without any power reduction technique), (ii) sleep/wake scheme, and (iii) the *SWORD* algorithm, by using a digital multimeter (MCP Lab Electronics/MT8045) and a storage oscilloscope (MCP Lab Electronics/DQ7042C). The DHT11 and soil moisture sensors consume 1.85 mA and 0.1 mA in active mode, respectively, and 0.01 mA in idle mode (i.e., no measurement value) at a supply voltage of 3.3 V.

The Atmega 328p was practically implemented under standalone condition to reduce the power consumption of the battery of the network nodes. The selection of the oscillator frequency value is crucial in the power consumption of the microcontroller, as presented in [42], where the Atmega 328p microcontroller consumed 6.25 mA at 16 MHz. A trade-off between power consumption and processing speed is necessary. Therefore, an operating frequency of 16 MHz is selected in the current experiment to minimize measurement time. In such case, the microcontroller Atmega 328p consumes 6.25 mA in active mode and 0.09 mA in power-saving mode [55].

**Figure 9:**
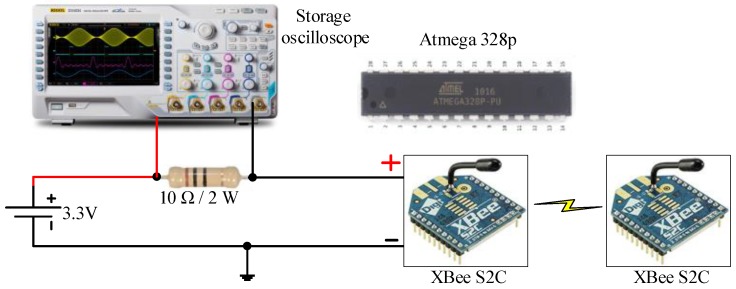
Test bed current consumption measurements of Atmega 328p and XBee S2C.

**Figure 10:**
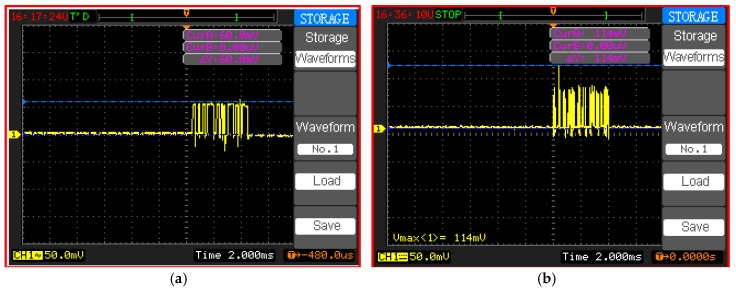
Active current consumption using a storage oscilloscope (DQ7042C) for (**a**) the Atmega328p microcontroller and (**b**) XBee S2C.

Our test bed measurements show that an active current consumption of 6 mA (Figure 9) is recorded for the microcontroller (Figure 10a). The active current drain of the Atmega 328p microcontroller is measured using an oscilloscope, whereas the power-saving mode is measured using a digital multimeter because a low current consumption value cannot be captured by an oscilloscope. In the test bed measurement, a 10 Ω shunt resistor is connected between the supply pin of the microcontroller and the voltage source (battery: 3.3 V). A small shunt resistor value is selected to reduce voltage loss in the supply line of the microcontroller. The drained current is obtained in milliamperes by dividing the measured voltage across the shunt resistor by the shunt resistor value of 10 Ω (I = V/R). Therefore, the active current consumption of Atmega 328p during the transmission process is 60 mV/10 Ω = 6 mA in active mode, as shown in Figure 10a.

In the WSN hardware, the main power is consumed by the RF module during data transmission and reception [56]. Therefore, the same procedure as that in the microcontroller measurements of active current consumption for XBee S2C can be applied. An active current consumption of 11.4 mA is measured during the transmission process, as shown in Figure 10b. However, the sleep current consumption of XBee S2C is measured to be 0.58 mA using the digital multimeter. The parameters in Equations (2)–(11) with and without the sleep/wake scheme for the sensor node are presented in Table 4. Furthermore, the parameters in Equations (12)–(15) for the router and main router nodes are provided in Table 4. The values in Table 4 are measured for active time, sleep time, DC, and active and sleep current consumption with and without the sleep/wake scheme.

### 9.2. Power Consumption Based on the Sleep/Wake Scheme

**Figure 11:**
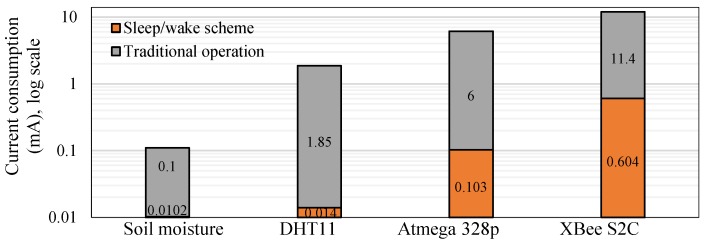
Current consumption of each sensor node component with and without the sleep/wake scheme.

Figure 11 shows the current consumption of each component of the sensor node with and without the sleep/wake scheme. The current consumption of XBee S2C is the highest among all the components of the sensor node because RF components frequently transmit data with maximum output power to ensure data delivery. 

**Figure 12:**
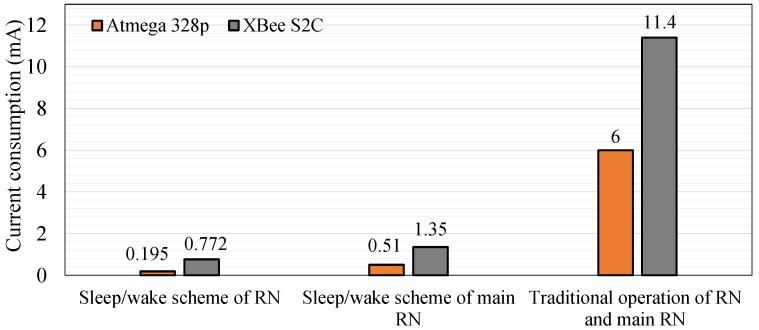
Current consumption of the components of the router and main router nodes with and without the sleep/wake scheme.

In the current work, +5 dBm (3.1 mW) is adopted to ensure communication between WSN nodes in the farm field. Figure 12 shows the power consumption of the router and main router nodes. The current consumption of the router nodes is considerably improved by the sleep/wake scheme. The power consumed by the router nodes is higher than that consumed by the sensor node (Table 4). This result is attributed to the following conditions:

(i) The router node collects climate condition data from four sensor nodes within 16 s, whereas the sensor node measures data using two sensors within 2 s.
(ii) The payload of the router node is higher than those of the sensor nodes, as indicated in Figure 4b,c.
(iii) The DC of the router node is larger than that of the sensor node, as shown in Figure 6.

**Figure 13:**
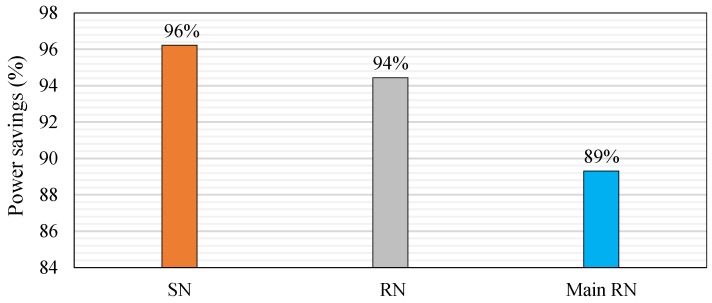
Developed power savings of the WSN nodes based on the sleep/wake scheme.

Figure 13 illustrates that when the sleep/wake scheme is applied, the power savings of the sensor node are considerably increased to 96% relative to that in traditional operation, which consumes 19.35 mA. Power saving is computed using Equation (17) [57]:

(17) $$\;Power\;savings=\left(1-\frac{current\;consumption_{sleep/wake\;scheme}}{current\;consumption_{traditional\;operation}}\right)\;\times \;100\%\;$$

In addition, power savings are improved to 90%, 99%, 98%, and 95% for soil moisture, DHT11, Atmega 328p, and XBee S2C relative to their values in traditional operation, respectively. Power savings are increased to 94% and 89% for the router and main router nodes, respectively (Figure 13), relative to traditional operation (i.e., without the sleep/wake scheme), which consumes 17.4 mA for the router and main router nodes. Power savings are computed using Equation (17). Figure 13 shows that the power savings of the sensor node are better than those of the router and main router nodes for the same reasons mentioned previously. The power saving of the router node is 96.7% and 93% for Atmega 328p and XBee S2C relative to traditional operation, respectively. The power saving of the main router is 91.5% and 88% for Atmega 328p and XBee S2C relative to traditional operation, respectively.

### 9.3. Battery Life Estimation Based on the Sleep/Wake Scheme

**Figure 14:**
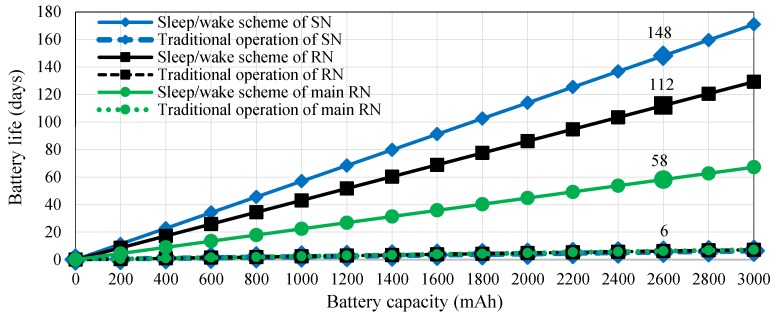
Estimated battery life versus battery capacity in the WAS based on the sleep/wake scheme and in the traditional operation.

The application of the sleep/wake scheme verifies that this approach can improve the power consumption of the proposed WAS to 0.731 (sensor node), 0.967 (router node), and 1.86 mA (main router node). Consequently, battery life can be extended to 148 (sensor node), 112 (router node), and 58 (main router node) days by using two rechargeable Li-ion battery with 7.4 V/2600 mAh. The battery life of the sensor node and the router/ main router nodes is 5.6 days and 6 days in the traditional operation, respectively. The battery life for a particular energy of the battery, which relies on Equation (11) at the current usage of the sensor, router, and main router nodes, is presented in the relationship in Figure 14. The figure also shows the improvement in the current drained by the nodes of the WAS when the sleep/wake scheme is considered.

### 9.4. Results of the SWORD Algorithm

**Figure 15:**
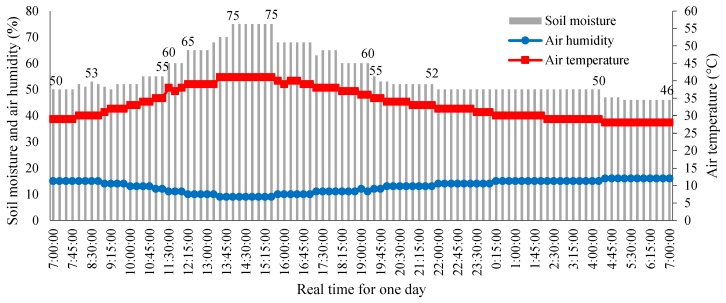
Measured and transmitted data about climate conditions from the sensor node to the router node.

The application of the *SWORD* algorithm depends on soil moisture measurements. Air temperature, air humidity, and soil moisture are measured by the sensor node of the WAS. Experiments are conducted several times on different days between June 2018 and July 2018 to measure the climate conditions in the farm. However, the measurements (Figure 15) performed on 8 July 2018 are considered in this study to verify the performance of the proposed *SWORD* algorithm because the weather conditions on that day are harsh (e.g., high air temperature and low soil humidity) in the considered area. The measurements are configured to capture data every 15 min, as seen in the timing diagram of the sleep/wake scheme in Figure 6. The sensor node transmits the climate conditions to the coordinator node every 15 min via the router and main router nodes. In this case, 96 samples (from 4 samples every hour) are collected in a day (Figure 15).

**Figure 16:**
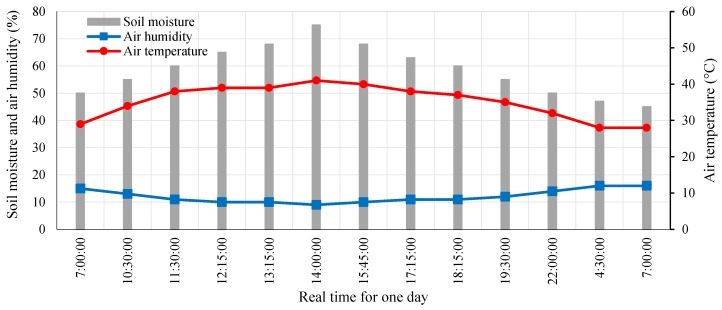
Measured and transmitted data regarding climate conditions from the sensor node to the router node by applying the *SWORD* algorithm.

The *SWORD* algorithm checks whether the measured data regarding soil moisture are redundant. When the difference between the preceding and subsequent soil moisture measurements is zero or less than or equal to 5%, the data are not transmitted to the router node. Otherwise, the data are transmitted to the router node. The transmitted data are minimized through this strategy. Thus, the power consumption of the sensor node is considerably improved relative to the sleep/wake scheme and traditional operation. In the *SWORD* algorithm, the transmitted data from the sensor node to the router node are minimized to 13 samples, as shown in Figure 16. Soil moisture measurements are weighted within the range of 45–75%. The reading range of 45–49% denotes dry soil, 50–55% represents moderate moist or field capacity, and 55–75% indicates wet or saturated soil. The *SWORD* algorithm minimizes data transmission (*data_tx*) by 86.45% relative to the sleep/wake scheme, as obtained using Equation (18):

(18) $$\;Minimization_{data\_tx}=\left(1-\frac{samples\;based\;on\;SWORD\;algorithm}{samples\;based\;on\;sleep/wake\;scheme}\right)\;\times \;100\%\;$$

Therefore, the average current consumption of the sensor node is remarkably improved to 0.1 mA by applying Equation (19). In this case, a power consumption of 0.74 mW can be dissipated during the transmission of climate conditions in the sensor node based on the *SWORD* algorithm:

(19) $$I_{SN\_avg\_SWORD}=I_{SN\_avg\;}-\;\left(\frac{Minimization_{data\_tx}}{100}\right)\;\times \;I_{SN\_avg},$$

where $I_{SN\_avg\;}$ is the average current consumption of 0.731 mA of the sensor node, which is previously computed using Equation (7) based on the application of the sleep/wake scheme.

**Figure 17:**
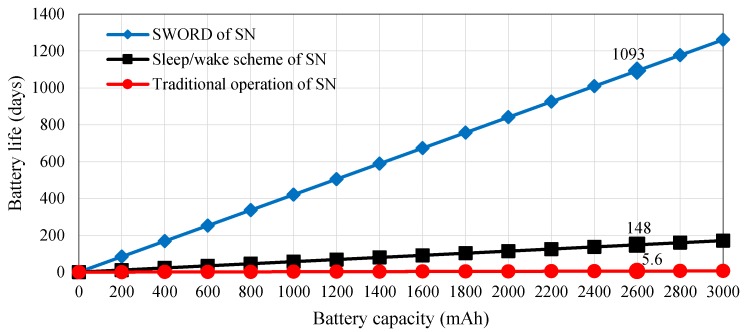
Estimated battery life versus battery capacity in the sensor node based on the *SWORD* algorithm, sleep/wake scheme, and traditional operation.

Consequently, battery life can be prolonged to 1093 days (3 years) by applying Equation (11) and using the same battery capacity of the sensor node (i.e., 7.4/2600 mAh). The battery life for a particular energy of the battery at the current usage of the sensor node is estimated in the relationship shown in Figure 17. The figure indicates that the power savings of the sensor node based on the *SWORD* algorithm considerably improve battery life to 86.45% and 99.48% relative to the sleep/wake scheme and traditional operation, respectively. However, these percentages may increase or decrease depending on the weather, soil conditions, and farm location. As weather temperature increases, soil condition changes rapidly from moisturized to dehydrated, thereby remarkably changing soil moisture measurements. Data transmission can occur, and power consumption increases. By contrast, data transmission and power consumption are considerably minimized when soil moisture is constant under good weather or rainfall conditions. During rainfall condition, the microcontroller measures soil moisture based on the soil humidity sensor to control the operation of the *SWORD* algorithm. In case of rainfall, the soil is wet, irrigation is unnecessary, and no data transmission occurs from the sensor node to the router node. Consequently, the sensors and XBee module of the sensor node enter sleep mode to conserve energy. In addition, power consumption and data transmission vary from summer to winter. However, soil moisture changes according to temperature and humidity. In our work, we set the constant value of the soil moisture (i.e., threshold) to trigger the sensor to alert the users if intervention is necessary, such as a switch on the water irrigation system or based on an automated irrigation system. The percentage values of 40–50% are considered acceptable soil moisture values in plant growing environments as proven in [58]. 

The output power of the solar panel is adequate to supply the sensor node because the power consumption of the sensor node varies from 0.75 (*SWORD* algorithm) and 5.409 (sleep/wake scheme) to 143.19 mW (traditional operation). This result is based on the recommendation in [26], which indicates that the capacity of a solar cell should be at least six times the average power consumption of the load. The power consumption of the router and main router nodes is 7.155 mW and 13.764 mW for the sleep/wake scheme, whereas the value is 128.76 mW for traditional operation. The energy of the adopted solar cell can successively supply the sensor, router, and main router nodes. Consequently, when the solar cell (12 V/5 W) is used with the WAS hardware to supply power to the nodes alongside the *SWORD* algorithm, an infinite energy supply for the WAS node can be expected.

## 10. Power Consumption Comparison

**Figure 18:**
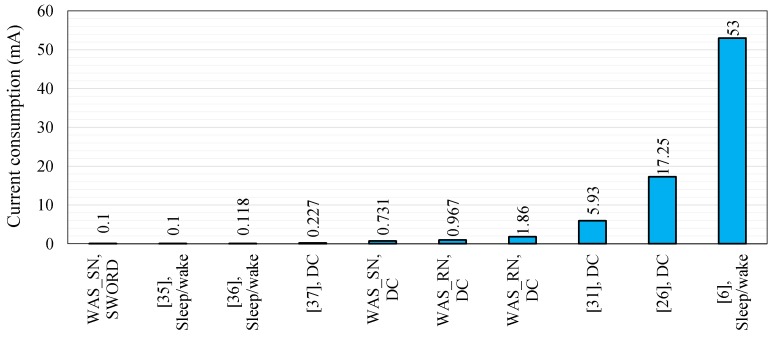
Current consumption of the proposed algorithms, together with the other energy efficient schemes used specifically in precision agriculture application.

The power consumption of the WAS using the sleep/wake scheme and the *SWORD* algorithm is compared with the power consumption of other schemes presented in existing studies on agriculture monitoring systems (Figure 18) to verify the proposed system. 

The performance of this work in terms of current consumption is achieved based on the methodology designed in Section 6, Section 7 and Section 8 and is validated by the actual hardware (prototype) implementation (Section 4) and on-site measurement (Section 9). The proposed algorithms are compared with those presented in related studies based on the values recorded during measurements in the previous literature. The performance in terms of average current consumption of previous methods is presented in detail in Section 2.

Although the previous works are based on energy efficient approaches, however, it is important to acknowledge that the measurement taken from this study might differ from the previous works in the literature, taken into account different factors, which include: (i) the sensors’ power requirements, (ii) techniques/algorithms introduced in the previous works and (iii) variation of the agriculture conditions (such as humidity, temperature and other environmental conditions) where the previous works have been deployed.

The sensor node current consumption of the proposed WAS is reduced based on two approaches: the first is sleep/wake scheme adopting DC and the second is *SWORD* algorithm. The current consumption of the sensor node (0.731 mA), router node (0.967 mA), and main router node (1.86 mA) are achieved based on sleep/wake scheme using low DC. We observe that the current consumption of the proposed WAS using the *SWORD* algorithm is approximately similar to the performance of the approaches in [35,36,37], which have achieved a current consumption of 0.1 (based on sleep/wake), 0.118 (based on sleep/wake), and 0.227 mA (based on DC), respectively. The average current consumption of our proposed WAS is 0.1 mA and 0.731 mA for the *SWORD* algorithm and the sleep/wake scheme, respectively, as shown in Figure 18.

## 11. Conclusions

We propose an energy-efficient scheme known as the WAS for agricultural application. The system is designed and practically implemented to monitor the climate conditions of crops, including air temperature, air humidity, and soil moisture. System hardware is carefully selected based on energy-efficient criteria to minimize the power consumption of different nodes in the WSN, such as the low-power Zigbee wireless protocol (i.e., XBee S2C), the standalone Atmega 328p microcontroller, and low-power sensors. In addition, two power reduction techniques are proposed to further improve the power consumption of the sensor, router, and main router nodes. These two schemes are the sleep/wake scheme and the *SWORD* algorithm. The sleep/wake scheme is achieved based on different DCs of the nodes in the WAS (i.e., 0.222% for the sensor node, 1.778% for the router node, and 7.111% for the main router node). The *SWORD* algorithm combines the sleep/wake scheme and the minimization of redundant data from sensor node packets. The power consumption of the sensor, router, and main router nodes is considerably improved when the two proposed methods are used.

The power saving and data communication achieved by applying the *SWORD* algorithm may be increased or decreased depending on the redundant data from soil moisture measurements, which depend on the soil condition (wet or dry). The power savings in the current work are improved by 86.45% and 99.48% relative to the sleep/wake scheme and traditional operation, respectively. In addition, data communication is minimized by 86.45%. The results of current consumption are compared with those in previous studies to validate the performance of the proposed system. The proposed WAS allows data collection for decision support in farming fields and can assist users in automating irrigation systems in agricultural fields in the future. The use of an energy-efficient and advanced WSN technology can achieve highly productive and sustainable precision farming.

## Figures and Tables

**Table 1:** Comparison between the current consumption of the proposed system and those of systems presented in previous research on agricultural applications.

Reference	Type of Microcontroller	Type of Wireless Protocol	Type of Sensor	Type of Battery Cell	Current/Voltage/Power of Solar Panel	Techniques for Reducing Power Consumption	Current or Power Consumption
[6]	8051	Zigbee-based CC2530	Temperature, humidity, moisture	Lead-acid 6 V/4500 mAh	9 V/5 W	Solar panel and DC (sleep)	53 mA
[25]	8051	Zigbee	Temperature, humidity	Lithium 3.7 V/2500 mAh	4 V/120 mA	Solar panel and DC	80 mA
[20]	MSP430F22x2, MSP430F22x4	RF module, CC2500	Temperature, humidity (SHT11)	AAA batteries 4.5 V/2000 mAh	N/A	Solar panel and DC (sleep mode)	0.142 mA
[26]	MSP430	Zigbee-based CC1120	Wind speed and direction, temperature, humidity, rain gauge, water, pH level	Lead-acid 12 V/4500 mAh	12 V/2 W	Solar panel and DC (sleep mode)	17.25 mA
[27]	H8 “VS-WRC003LV”	802.11a	Temperature, humidity	NiCad 2.4 V/1000 mA	180 mW (sunny) 24 mW (shady)	Solar panel and DC	11.11 mA
[28]	PCI32MX220F032B	Wi-Fi/Zigbee (IEEE 802.15.4)	Temperature, pH, DO	N/A	N/A	Solar panel and DC	81.5 mA
[29]	MEGA 2560	XBee	Passive infrared sensor	Lead-acid 6 V/4.5 Ah	4.75 V	Solar panel and DC (sleep mode)	27 Wh
[31]	N/A	Zigbee and GSM/GPRS	Moisture, temperature, pressure, water conductivity	Lithium-ion 3.7 V/1900 mAh	5 V/0.8 W 160 mA	Solar panel and DC (sleep mode)	5.93 mA
[24]	N/A	CC1110	Temperature, humidity	3.7 V/850 mAh	500 mW	Solar panel and DC (sleep mode)	4.427 mA
[32]	N/A	nRF24L01	pH	Not used	N/A	DC (sleep mode)	2.807 mA
[33]	Atmega 324P	LoRa	Soil temperature and moisture, air temperature and humidity, light intensity	NI-MH AA 2.4 V/2400 mAh	N/A	DC (sleep mode)	0.544 mA
[34]	N/A	XBee PRO Series 2	Soil moisture, volumetric water content	Lithium	12 V/10 W	Solar panel and DC (sleep mode)	177 mA (active mode) 3.5 µA (sleep mode)
[35]	MSP430F1611	IEEE 802.15.4 (CC2420)	Air temperature and soil moisture	NiMH 4 V/2700 mAh	12 V/6.5 Ah	Solar panel and sleep/wake scheme (MAC protocol)	0.1 mA
[36]	MSP430F149	IEEE 802.15.4 (CC2420)	Temperature, light intensity, and humidity	Lithium 2.7 V/2000 mAh	N/A	Sleep/wake scheme (MAC protocol)	0.118 mA
[37]	8051	Zigbee (CC2530)	Soil temperature and moisture, temperature, and humidity	Lithium-ion 7 V/1000 mAh	7 V/7 W	Solar panel and DC (sleep mode)	0.227 mA

DO: dissolved oxygen.

**Table 2:** Major components of the sensor node for the WAS hardware.

Number	Hardware Type	Description
1	Temperature and humidity sensor	DHT11
2	Soil moisture sensor	YL-100
3	Microcontroller	Atmega 328p as standalone
4	Wireless protocol	Zigbee based on XBee S2C [40,41]
5	Power systems	Li-ion battery (7.4 V/2600 mAh)
6	Power solar cell	KINGRO-004V (12 V/5 W)
7	DC–DC converter	LM 2596
8	Charger controller	PWM-LS2024E

**Table 3:** Characteristics of the adopted solar cell.

Parameter	Value
Output voltage	12 V
Load voltage	11.1 V
Maximum current	416 mA
Maximum output power (*P_max_*)	5 W
Dimension (L × W × T)	185 × 285 × 3.2 mm^3^
Surface area (S)	527.25 cm^2^

**Table 4:** Current consumption and time profile of the sensor, router, and main router nodes.

	Components of Sensor Node				Components of Router Node		Components of Main Router Node	
Parameters	Soil Moisture	DHT11	Atmega 328p	XBee S2C	Atmega 328p	XBee S2C	Atmega 328p	XBee S2C
*I_active_* (mA)	0.1	1.85	6	11.4	6	11.4	6	11.4
*I_sleep_* (mA)	0.01	0.01	0.09	0.58	0.09	0.58	0.09	0.58
*t_active_* (s)	2	2	2	2	16	16	16	16
*t_sleep_* (s)	898	898	898	898	884	884	884	884
*T_total_* (s)	900	900	900	900	900	900	900	900
DC	0.2222%	0.2222%	0.2222%	0.2222%	1.778%	1.778%	7.111%	7.111%
*I_avg_* (mA)	0.0102 Equation (3)	0.0140 Equation (4)	0.103 Equation (5)	0.604 Equation (6)	0.195 Equation (5)	0.772 Equation (6)	0.51 Equation (5)	1.35 Equation (6)
*I* (mA)	19.35 Equation (2)				17.4 Equation (12)		17.4 Equation (12)	
*I_avg_* (mA)	0.731 Equation (7)				0.967 Equation (13)		1.86 Equation (13)	
*I_total_* (mA)	309.6 Equation (8)				69.6 Equation (14)		17.4 Equation (14)	
*I_avg_total_* (mA)	11.703 Equation (9)				3.869 Equation (15)		1.86 Equation (15)	
*P_avg_* = *I_avg_* × V based on sleep/wake (mW)	*P_avg_SN_* = 7.4 × 0.731 = 5.409 Equation (10)				*P_avg_RN_* =7.4 × 0.967 = 7.155 Equation (10)		*P_avg_RN_* = 7.4 × 1.86 = 13.764 Equation (10)	
*P* (mW) = I × V without sleep/wake	*P* = I × V = 7.4 V × 19.35 mA = 143.19				*P* = 7.4 × 17.4 =128.76		*P* = 7.4 × 17.4 =128.76	
*L_life_* with sleep/wake (days)	148 (3554 h) Equation (11)				112 (2687 h) Equation (11)		58 (1398 h) Equation (11)	
*L_life_* without sleep/wake (days)	5.6 (134 h) Equation (11)				6 (149 h) Equation (11)		6 (149 h) Equation (11)	

## References

[1] Kim Y.-D., Yang Y.-M., Kang W.-S., Kim D.-K. (2014). On the design of beacon based wireless sensor network for agricultural emergency monitoring systems. Comput. Stand. Interfaces.

[2] Yang J., Zhou J., Lv Z., Wei W., Song H. (2015). A real-time monitoring system of industry carbon monoxide based on wireless sensor networks. Sensors.

[3] Wu T., Wu F., Redouté J.-M., Yuce M.R. (2017). An autonomous wireless body area network implementation towards iot connected healthcare applications. IEEE Access.

[4] Erd M., Schaeffer F., Kostic M., Reindl L.M. (2016). Event monitoring in emergency scenarios using energy efficient wireless sensor nodes for the disaster information management. Int. J. Disaster Risk Reduct..

[5] Rawat P., Singh K.D., Chaouchi H., Bonnin J.M. (2014). Wireless sensor networks: A survey on recent developments and potential synergies. J. Supercomput..

[6] Zhu B., Han W., Wang Y., Wang N., Chen Y., Guo C. (2014). Development and evaluation of a wireless sensor network monitoring system in various agricultural environments. J. Microw. Power Electromagn. Energy.

[7] Srbinovska M., Gavrovski C., Dimcev V., Krkoleva A., Borozan V. (2015). Environmental parameters monitoring in precision agriculture using wireless sensor networks. J. Clean. Prod..

[8] Mesas-Carrascosa F., Santano D.V., Meroño J., de la Orden M.S., García-Ferrer A. (2015). Open source hardware to monitor environmental parameters in precision agriculture. Biosyst. Eng..

[9] Azaza M., Tanougast C., Fabrizio E., Mami A. (2016). Smart greenhouse fuzzy logic based control system enhanced with wireless data monitoring. ISA Trans..

[10] Aiello G., Giovino I., Vallone M., Catania P., Argento A. (2018). A decision support system based on multisensor data fusion for sustainable greenhouse management. J. Clean. Prod..

[11] Nikolidakis S.A., Kandris D., Vergados D.D., Douligeris C. (2015). Energy efficient automated control of irrigation in agriculture by using wireless sensor networks. Comput. Electron. Agric..

[12] Ojha T., Misra S., Raghuwanshi N.S. (2015). Wireless sensor networks for agriculture: The state-of-the-art in practice and future challenges. Comput. Electron. Agric..

[13] Jawad H.M., Nordin R., Gharghan S.K., Jawad A.M., Ismail M. (2017). Energy-efficient wireless sensor networks for precision agriculture: A review. Sensors.

[14] Wamuyu P.K. (2017). A conceptual framework for implementing a wsn based cattle recovery system in case of cattle rustling in kenya. Technologies.

[15] Nadimi E.S., Jørgensen R.N., Blanes-Vidal V., Christensen S. (2012). Monitoring and classifying animal behavior using zigbee-based mobile ad hoc wireless sensor networks and artificial neural networks. Comput. Electron. Agric..

[16] Huircán J.I., Muñoz C., Young H., Von Dossow L., Bustos J., Vivallo G., Toneatti M. (2010). Zigbee-based wireless sensor network localization for cattle monitoring in grazing fields. Comput. Electron. Agric..

[17] Kwong K.H., Wu T.-T., Goh H.G., Sasloglou K., Stephen B., Glover I., Shen C., Du W., Michie C., Andonovic I. (2012). Practical considerations for wireless sensor networks in cattle monitoring applications. Comput. Electron. Agric..

[18] Raheemah A., Sabri N., Salim M., Ehkan P., Ahmad R.B. (2016). New empirical path loss model for wireless sensor networks in mango greenhouses. Comput. Electron. Agric..

[19] Pan H., Shi Y., Wang X., Li T. (2017). Modeling wireless sensor networks radio frequency signal loss in corn environment. Multimedia Tools Appl..

[20] Srbinovska M., Dimcev V., Gavrovski C. (2017). Energy Consumption Estimation of Wireless Sensor Networks in Greenhouse Crop Production. Proceedings of the 17th International Conference on Smart Technologies.

[21] Sahota H., Kumar R., Kamal A. (2011). A wireless sensor network for precision agriculture and its performance. Wirel. Commun. Mob. Comput..

[22] Sinha K., Sinha B.P., Datta D. (2011). An energy-efficient communication scheme for wireless networks: A redundant radix-based approach. IEEE Trans. Wirel. Commun..

[23] Kamarudin L.M., Ahmad R.B., Ndzi D.L., Zakaria A., Kamarudin K., Ahmed M.E.E.S. (2016). Simulation and analysis of leach for wireless sensor networks in agriculture. Int. J. Sens. Netw..

[24] De la Concepcion A.R., Stefanelli R., Trinchero D. (2014). A Wireless Sensor Network Platform Optimized for Assisted Sustainable Agriculture. Proceedings of the IEEE Global Humanitarian Technology Conference (GHTC).

[25] Zou T., Lin S., Feng Q., Chen Y. (2016). Energy-efficient control with harvesting predictions for solar-powered wireless sensor networks. Sensors.

[26] Nguyen T.-D., Thanh T.T., Nguyen L.-L., Huynh H.-T. On the Design of energy Efficient Environment Monitoring Station and Data Collection Network Based on Ubiquitous Wireless Sensor Networks. Proceedings of the 2015 IEEE RIVF International Conference on Computing & Communication Technologies-Research, Innovation, and Vision for the Future (RIVF).

[27] Eto M., Katsuma R., Tamai M., Yasumoto K. Efficient coverage of agricultural field with mobile sensors by predicting solar power generation. Proceedings of the 2015 IEEE 29th International Conference on Advanced Information Networking and Applications (AINA).

[28] Fourie C., Bhatt D., Silva B., Kumar A., Hancke G. A solar-powered fish pond management system for fish farming conservation. Proceedings of the International Symposium on Industrial Electronics (ISIE).

[29] Bapat V., Kale P., Shinde V., Deshpande N., Shaligram A. (2017). Wsn application for crop protection to divert animal intrusions in the agricultural land. Comput. Electron. Agric..

[30] Villarrubia G., Paz J.F.D., Iglesia D.H., Bajo J. (2017). Combining multi-agent systems and wireless sensor networks for monitoring crop irrigation. Sensors.

[31] Navarro-Hellín H., Torres-Sánchez R., Soto-Valles F., Albaladejo-Pérez C., López-Riquelme J.A., Domingo-Miguel R. (2015). A wireless sensors architecture for efficient irrigation water management. Agric. Water Manag..

[32] Cambra C., Sendra S., Lloret J., Lacuesta R. (2018). Smart system for bicarbonate control in irrigation for hydroponic precision farming. Sensors.

[33] Ilie-Ablachim D., Pătru G.C., Florea I.-M., Rosner D. (2016). Monitoring device for culture substrate growth parameters for precision agriculture: Acronym: Monisen. Proceedings of the 2016 15th RoEduNet Conference: Networking in Education and Research.

[34] Zaier R., Zekri S., Jayasuriya H., Teirab A., Hamza N., Al-Busaidi H. Design and implementation of smart irrigation system for groundwater use at farm scale. Proceedings of the 2015 7th International Conference on Modelling, Identification and Control (ICMIC).

[35] López J.A., Garcia-Sanchez A.-J., Soto F., Iborra A., Garcia-Sanchez F., Garcia-Haro J. (2011). Design and validation of a wireless sensor network architecture for precision horticulture applications. Precis. Agric..

[36] Sun T., Yan X.J., Yan Y. (2013). A chain-type wireless sensor network in greenhouse agriculture. J. Comput..

[37] Mittal A., Chetan K., Jayaraman S., Jagyasi B.G., Pande A., Balamuralidhar P. (2012). Mkrishi wireless sensor network platform for precision agriculture. Proceedings of the Sixth International Conference on Sensing Technology (ICST).

[38] Granda-Cantuña J., Molina-Colcha C., Hidalgo-Lupera S.-E., Valarezo-Varela C.-D. (2018). Design and implementation of a wireless sensor network for precision agriculture operating in api mode. Proceedings of the International Conference on eDemocracy & eGovernment (ICEDEG).

[39] Ndzi D.L., Harun A., Ramli F.M., Kamarudin M.L., Zakaria A., Shakaff A.Y.M., Jaafar M.N., Zhou S., Farook R.S. (2014). Wireless sensor network coverage measurement and planning in mixed crop farming. Comput. Electron. Agric..

[40] Chen Y.-C., Chen P.-Y., Wen C.-Y. (2016). Request-centric wireless bus information management system. Inventions.

[41] Ante M.G.E., Garcia K.A.N., Gonzales B.A.A., Mendoza J.T.D., Roque M.A.C. (2018). Microcontroller-based power monitoring and switching device for appliances over a zigbee network. J. Telecommun. Electron. Comput. Eng..

[42] Gharghan S.K., Nordin R., Ismail M. (2015). An ultra-low power wireless sensor network for bicycle torque performance measurements. Sensors.

[43] Xbee/Xbee-Pro RF Modules. Http://www.Digi.Com.

[44] Xbee®/Xbee-Pro s2c Zigbee®rf Module. Https://www.Digi.Com/resources/documentation/digidocs/pdfs/90002002.Pdf.

[45] Lian K.-Y., Hsiao S.-J., Sung W.-T. (2013). Intelligent multi-sensor control system based on innovative technology integration via zigbee and wi-fi networks. J. Netw. Comput. Appl..

[46] Liu A., Chen Z., Xiong N.N. (2018). An adaptive virtual relaying set scheme for loss-and-delay sensitive wsns. Inf. Sci..

[47] Kulatunga C., Shalloo L., Donnelly W., Robson E., Ivanov S. (2017). Opportunistic wireless networking for smart dairy farming. IT Prof..

[48] Ivanov S., Bhargava K., Donnelly W. (2015). Precision farming: Sensor analytics. IEEE Intell. Syst..

[49] Navarro-Hellín H., Martínez-del-Rincon J., Domingo-Miguel R., Soto-Valles F., Torres-Sánchez R. (2016). A decision support system for managing irrigation in agriculture. Comput. Electron. Agric..

[50] Soulis K.X., Elmaloglou S., Dercas N. (2015). Investigating the effects of soil moisture sensors positioning and accuracy on soil moisture based drip irrigation scheduling systems. Agric. Water Manag..

[51] Gharghan S.K., Nordin R., Ismail M. (2014). Energy-efficient zigbee-based wireless sensor network for track bicycle performance monitoring. Sensors.

[52] Guo S., Wang C., Yang Y. (2014). Joint mobile data gathering and energy provisioning in wireless rechargeable sensor networks. IEEE Trans. Mob. Comput..

[53] Escolar S., Chessa S., Carretero J. (2014). Energy management in solar cells powered wireless sensor networks for quality of service optimization. Pers. Ubiquitous Comput..

[54] Visconti P., Primiceri P., Orlando C. (2016). Solar powered wireless monitoring system of environmental conditions for early flood prediction or optimized irrigation in agriculture. J. Eng. Appl. Sci..

[55] Atmel Inc. Atmel 8-bit Avr Microcontroller with 4/8/16/32k Bytes in System Programmable Flash. http://www.atmel.com/Images/doc8161.pdf.

[56] Magno M., Marinkovic S., Srbinovski B., Popovici E.M. (2014). Wake-up radio receiver based power minimization techniques for wireless sensor networks: A review. Microelectron. J..

[57] Gharghan S.K., Nordin R., Ismail M. (2016). Energy efficiency of ultra-low-power bicycle wireless sensor networks based on a combination of power reduction techniques. J. Sens..

[58] Ramlow M., Cotrufo M.F. (2018). Woody biochar’s greenhouse gas mitigation potential across fertilized and unfertilized agricultural soils and soil moisture regimes. GCB Bioenergy.

